# Parallel phonological processing of Chinese characters revealed by flankers tasks

**DOI:** 10.3389/fpsyg.2023.1239256

**Published:** 2023-10-06

**Authors:** Ruifeng Yu, Yunong Wu, Feng Gu

**Affiliations:** ^1^Neurocognitive Laboratory for Linguistics and Semiotics, College of Literature and Journalism, Sichuan University, Chengdu, China; ^2^Digital Convergence Laboratory of Chinese Cultural Inheritance and Global Communication, Sichuan University, Chengdu, China; ^3^Rollins School of Public Health, Emory University, Atlanta, GA, United States

**Keywords:** parallel phonological processing, Chinese characters, flankers tasks, logographic scripts, reading

## Abstract

An important and extensively researched question in the field of reading is whether readers can process multiple words in parallel. An unresolved issue regarding this question is whether the phonological information from foveal and parafoveal words can be processed in parallel, i.e., parallel phonological processing. The present study aims to investigate whether there is parallel phonological processing of Chinese characters. The original and the revised flankers tasks were applied. In both tasks, a foveal target character was presented in isolation in the no-flanker condition, flanked on both sides by a parafoveal homophone in the homophone-flanker condition, and by a non-homophonic character in the unrelated-flanker condition. Participants were instructed to fixate on the target characters and press two keys to indicate whether they knew the target characters (lexical vs. non-lexical). In the original flankers task, the stimuli were presented for 150 ms without a post-mask. In the revised flankers task, we set the stimulus exposure time (duration of the stimuli plus the blank interval between the stimuli and the post-mask) to each participant’s lexical decision threshold to prevent participants from processing the target and flanker characters serially. In both tasks, reaction times to the lexical targets were significantly shorter in the homophone-flanker condition than in the unrelated-flanker condition, suggesting parallel phonological processing of Chinese characters. In the revised flankers task, accuracy rates to the lexical targets were significantly lower in the unrelated-flanker condition compared to the homophone-flanker condition, further supporting parallel phonological processing of Chinese characters. Moreover, reaction times to the lexical targets were the shortest in the no-flanker condition in both tasks, reflecting the attention distribution over both the target and flanker characters. The findings of this study provide valuable insights into the parallel processing mechanisms involved in reading.

## 1. Introduction

When communicating verbally, we can only perceive words one at a time, whereas during reading, many words appear in our visual field simultaneously. This raises an important and extensively researched question: can we process multiple words in parallel during reading (Engbert et al., 2002, 2005; Reichle et al., 2003, 2006, 2009; Richter et al., 2006; Schotter et al., 2012; Snell et al., 2018c; Snell and Grainger, 2019; White et al., 2019a)? This question not only concerns the mechanisms involved in reading but also the domains of vision and attention in general. Phonology is an intrinsic and critical constituent of written words. To date, however, whether the phonological information from foveal and parafoveal words can be processed in parallel, i.e., parallel phonological processing, remains unclear. The current study aims to employ flankers tasks to investigate whether there is parallel phonological processing of Chinese characters.

Previous studies investigating parallel processing of visual words can be classified into two categories based on the types of tasks employed. One category used sentence reading tasks (e.g., Angele et al., 2013, 2015; Brothers et al., 2017; Pan et al., 2021; Snell et al., 2023; Zang et al., 2023). In these studies, the observed parafoveal-on-foveal effects (POF effects), i.e., the influence of parafoveal words on the processing of foveal words, were generally considered as evidence of parallel word processing (Kennedy and Pynte, 2005; Zang et al., 2023), although some researchers have argued that POF effects could also be accounted for by mislocated fixations (Drieghe et al., 2008; Drieghe, 2011). The other category employed visual word recognition tasks (e.g., Dare and Shillcock, 2013; Snell et al., 2017a,b, 2019, 2021; White et al., 2018, 2019b, 2020; Cauchi et al., 2020; Kobayashi and Ogawa, 2020; Meade et al., 2021). Among these tasks, the flankers task has been employed quite widely (e.g., Dare and Shillcock, 2013; Grainger et al., 2014; Snell et al., 2017c, 2019, 2021; Kobayashi and Ogawa, 2020; Meade et al., 2021. In the classic flankers task, participants were presented with a foveal target stimulus (e.g., a letter or a number) surrounded by flanking stimuli and were asked to respond to the target while disregarding the flankers (Eriksen et al., 1973; Eriksen and Eriksen, 1974). In the flankers task used to investigate parallel word processing, the foveal target word and the parafoveal flankers were simultaneously exposed for approximately 150 ms. Researchers have argued that within such a brief stimulus exposure time, participants could only attend to and complete the processing of the target word (Snell et al., 2017b; Snell and Grainger, 2018, 2019). Thus, if participants’ reaction times or accuracy rates to the target word were influenced by the flankers, the flankers must be processed during rather than after target word processing, reflecting parallel processing of the target and the flankers. Notably, the influence of the parafoveal flankers on the processing of the foveal target word is also a POF effect.

Many studies employing the flankers task have shown that lexical decisions to the target words were facilitated by orthographically related flankers, reflecting parallel orthographic processing (Dare and Shillcock, 2013; Grainger et al., 2014; Snell et al., 2017c, 2019, 2021). For instance, lexical decisions were faster when the flankers were bigrams in the target word (e.g., RO ROCK CK) compared with when the flankers did not share any letters with the target word (e.g., ST ROCK EN) (Grainger et al., 2014). Parallel orthographic processing was also supported by several studies using sentence reading tasks, in which the orthographic POF effects were observed (Inhoff et al., 2000; Pynte et al., 2004; Starr and Inhoff, 2004; White, 2008). For instance, Inhoff and his colleagues found that participants spent more time viewing the target word when the post-target preview was in upper case compared with lower case (Inhoff et al., 2000). Besides parallel orthographic processing, the flankers task has also revealed parallel semantic and syntactic processing (Snell et al., 2017a,b; Meade et al., 2021). For instance, participants exhibited shorter reaction times when the target words were flanked by syntactically congruent stimuli compared to incongruent stimuli (Snell et al., 2017b). Nevertheless, parallel semantic or syntactic processing was not consistently observed in sentence reading tasks (Staub et al., 2007; Yan et al., 2009; Schotter et al., 2012; Snell et al., 2023; Zang et al., 2023), and this lack of consistency might partly be attributed to variations in the techniques and measurements employed. For example, Staub et al.’s eye-tracking study did not observe the syntactic POF effect during sentence reading (Staub et al., 2007), whereas a recent study combining eye-tracking with EEG found that although the syntactic POF effect was not revealed by eye-tracking data, it was revealed by brain potentials at approximately 100 ms after the foveal word fixation onset (Snell et al., 2023).

Interestingly, though parallel orthographic, semantic, and syntactic processing was observed using the flankers task, parallel phonological processing was not. Cauchi and his colleagues used French words as stimuli and found no significant phonological POF effect in the flankers task. In particular, lexical decision times to the target words (e.g., rose) were not significantly different between the pseudohomophone flanker condition (e.g., roze rose roze) and the orthographic-control flanker condition (e.g., rone rose rone) (Cauchi et al., 2020). The authors argued that the absence of the phonological POF effect was due to the distinct spatiotopic locations of the target and flanker words. Specifically, the phonological processing of a certain word was tied to its specific spatiotopic location. Therefore, even though the phonological information of the target and the flankers was processed in parallel, their phonological information could not be spatially integrated, resulting in an absence of the phonological POF effect. However, we contend that two additional problems should be considered. Firstly, the targets were real words while the flankers were pseudowords. Previous studies have shown that participants spent more time processing pseudowords than real words, known as the lexicality effect (e.g., Lv and Wang, 2012; Schuster et al., 2015; Gao et al., 2022; Guo et al., 2022; Marchezini et al., 2022). Therefore, in Cauchi’s study, the stimulus duration (170 ms) was likely only sufficient for participants to process the phonological information of the targets but not the flankers. Secondly, the orthographic-control flankers (e.g., rone) overlapped phonologically with the target words (e.g., rose). Because the investigation of the phonological POF effect involved a comparison between the pseudohomophone condition and the orthographic-control condition, the overlapped phonological information between the targets and the orthographic-control flankers would dilute the measurement of this effect (Vasilev et al., 2019).

The two problems in Cauchi et al.’s study stem from the strong connection between orthography and phonology in alphabetic scripts. For alphabetic words, the orthographic information indicates the pronunciation. Because of this, in many cases, two homophonic alphabetic words would orthographically overlap (e.g., “meet” and “meat”), hence the orthographic-control condition to disentangle the contribution of phonology from orthography in Cauchi et al.’s study. Moreover, since it might be difficult to find enough real words serving as orthographic-control words, all the flanker words in Cauchi et al.’s study were more readily available pseudowords. The use of pseudowords and the fact that the orthographic-control flankers (e.g., rone) overlapped phonologically with the targets (e.g., rose), as mentioned above, may lead to the absence of the phonological POF effect. Comparatively, these two problems can be easily addressed when investigating the parallel phonological processing of logographic scripts such as Chinese. For Chinese, the connection between orthography and phonology is less explicit relative to alphabetic scripts. Chinese employs Chinese characters (each Chinese character corresponds to one syllable and usually corresponds to a morpheme) to indicate meaning rather than a few letters to indicate pronunciation. High-frequency homophonic Chinese characters can be orthographically distinct (e.g., 对/dui4/−队/dui4/). Therefore, in the flankers task, the homophonic flanker characters can be of high-frequency and bear no orthographic resemblance to the target characters, addressing the two problems in Cauchi et al.’s study. In the present study, we investigated the parallel phonological processing of Chinese characters using the flankers task. Lexical decision times to the target characters were compared in three conditions: a no-flanker condition, a homophone-flanker condition (target and flanker characters were homophones), and an unrelated-flanker condition (target and flanker characters were phonologically distinct). Importantly, we ensured that all the target and flanker characters were high-frequency characters, and all the flanker characters were orthographically distinct from the target characters. If there is parallel phonological processing of Chinese characters, a significant phonological POF effect should be observed, i.e., lexical decision times to the foveal target characters should be significantly shorter when the parafoveal flankers are homophones compared to phonologically distinct characters. Moreover, as suggested by previous research (Snell et al., 2017c), a no-flanker condition was included to investigate the effects of flanker presence, which is helpful for further understanding the mechanisms underlying the parallel processing of Chinese characters.

Notably, other researchers (White et al., 2018, 2020; Brothers, 2022) have raised two experimental concerns regarding the original flankers task. Firstly, the stimulus duration (~150 ms) might be long enough to allow participants to process the target and the flankers serially. Secondly, due to the lack of a post-mask following the stimulus presentation, there would be short memory traces of the stimuli after the stimulus disappearance. Thus, participants might process the target and the flankers serially relying on those short-term memory traces. To address these two concerns, we used a revised flankers task adopted from White et al. (2018, 2019a,b, 2020). In the revised flankers task, the stimulus exposure time was reduced to 12 ms. After the stimulus presentation, there was a blank ISI followed by a post-mask composed of six “#” (######). Importantly, for each participant, the duration of the blank ISI was adjusted using a staircase procedure and was set to his/her lexical decision threshold. Moreover, the post-mask following the ISI would terminate the short-term memory traces of the stimuli. Therefore, in the revised flankers task, participants were unable to process the target and the flankers serially. To investigate whether the two concerns regarding the original flankers task led to the serial processing of the target and the flankers, the present study used both the original and the revised flankers tasks. If the phonological POF effect is observed only in the original flankers task, it would suggest that the results of previous research using the original flankers task reflected serial word processing rather than parallel word processing.

## 2. Methods

### 2.1. Participants

Thirty native Chinese readers (11 males and 19 females, aged 18–25 years, mean age = 21 years; SD = 2.2 years) were recruited. All participants were students from Sichuan University who had normal or corrected-to-normal vision and were without a history of reading difficulties and neurological disorders. Furthermore, all participants were right-handed assessed by the Edinburgh Inventory (Oldfield, 1971). Prior to the experiment, written consent forms were obtained from each participant. The Ethics Committee of Sichuan University provided ethical approval for the study.

### 2.2. Stimuli

We selected 120 high-frequency characters as foveal target characters (mean character frequency = 5.29 Zipf; mean stroke member = 6.56). The calculation of Zipf values equals log_10_ (frequency per billion characters) (van Heuven et al., 2014). Character frequency was based on the SUBTLEX-CH frequency list (Cai and Brysbaert, 2010). Besides, we paired each high-frequency target with a homophone-flanker character (i.e., homophone-flanker condition), and an unrelated-flanker character which was phonologically distinct from the target (i.e., unrelated-flanker condition). Both the homophone-flanker and unrelated-flanker characters bore no orthographic resemblance to the target character. Specifically, there were no shared components (components are building units and independent parts of Chinese characters) between any target and its corresponding (homophone and unrelated) flankers. Moreover, any target and its corresponding flanker character (homophonic or unrelated) could not form a two-character word. The flanker characters in the homophone-flanker condition and the unrelated-flanker condition were matched in the following properties: (a) Character frequency (mean character frequency for homophone flankers = 5.30 Zipf; mean character frequency for unrelated flankers = 5.37 Zipf). A paired-samples t-test showed no significant difference between the two groups of flankers in character frequency [*t* (119) = 1.296, *p* = 0.197, 2-tailed)]. (b) Visual complexity indexed by stroke number (stroke number refers to the numerical count of the lines taken to form a character; mean stroke number for homophone flankers = 6.80; mean stroke number for unrelated flankers = 6.55). A paired-samples *t*-test showed no significant difference between the two groups of flankers in stroke number [*t* (119) =1.951, *p* = 0.053, 2-tailed)]. (c) Visual structure. Visual structure refers to the way components are arranged within a character. The homophone-flanker character and the unrelated-flanker character paired with the same target character were of the same visual structure. (d) Low-level visual similarity with the targets. We calculated the low-level visual similarity between the targets and the flankers based on a website that generates an image similarity quotient.^1^ A paired-samples t-test showed that the visual similarity between the targets and the flankers did not significantly differ between the homophone-flanker and unrelated-flanker conditions [*t* (119) = −0.018, *p* = 0.985, 2-tailed)].

We also selected 360 rarely-used characters, 120 serving as targets and 240 serving as flankers. In the following description, we would refer to the rarely-used characters as non-lexical characters, because they were unknown (meaningless and unpronounceable) to the participants and thus not stored in the participants’ mental lexicon. Correspondingly, those high-frequency target and flanker characters would be referred to as lexical characters. The 240 non-lexical flanker characters were evenly divided into two groups, so that similar to the lexical target characters, each non-lexical target character was paired with two non-lexical flankers. Moreover, visual complexity (indexed by stroke number) of the non-lexical characters was matched with that of the lexical characters. The reason why we used rarely-used characters rather than pseudo-characters as non-lexical stimuli was that there did not exist the print versions of Chinese pseudo-characters in electronic font libraries. The print versions of pseudowords in alphabetic languages could be easily created by combing letters, whereas creating the print versions of Chinese pseudo-characters is a rather complex process which needs professional font design.

For the lexical characters, there were three experimental conditions: no-flanker, homophone-flanker, and unrelated-flanker conditions. Each condition contained 120 trials. For the non-lexical characters, because they were unpronounceable to the participants, there were only two experimental conditions: no-flanker and unrelated-flanker conditions. The no-flanker condition contained 120 trials, while the unrelated-flanker condition contained 240 trials. Table 1 provides an overview of each experimental condition (see Table 1 in Supplementary material for a full list of stimuli).

**Table 1:** Examples for each experimental condition.

Conditions		Left flanker	Central target	Right flanker
Lexical	No-flanker		对(/dui4/, right)	
	Homophone-flanker	队(/dui4/, team)	对(/dui4/, right)	队(/dui4/, team)
	Unrelated-flanker	收(/shou1/, receive)	对(/dui4/, right)	收(/shou1/, receive)
Non-lexical	No-flanker		冱	
	Unrelated-flanker	佧	冱	佧

The lexical characters are presented with their corresponding pronunciation in Chinese pinyin and their lexical meaning in parentheses.

### 2.3. Apparatus

E-prime 3.0 was applied to display the stimuli and record participants’ behavioral data. Stimuli were presented against a white background, on a 24-in monitor (Dell G2722HS) with a resolution of 1920 × 1,080 pixels and a refresh rate of 165 Hz. The viewing distance was 100 cm, with each character subtending approximately 1.6 degrees of visual angle, and the distance between the target character and the flanker character (center to center) subtending approximately 2.9 degrees of visual angle. The stimuli were displayed in Heiti font.

### 2.4. Procedure

**Figure 1:**
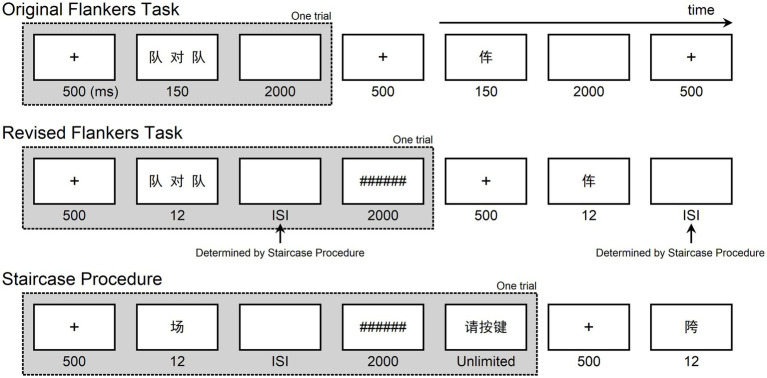
Illustration of the experimental procedures. In the two flankers tasks, participants were asked to press two keys to indicate whether they knew the target character as accurately and quickly as possible. In the staircase procedure, the duration of ISI varied following the designed algorithm to estimate the ISI threshold for the lexical decision.

The original and the revised flankers tasks were used (Figure 1). The order of the two tasks was counterbalanced across participants, and a short rest was provided between the two tasks. For each participant, the entire experiment ran approximately 90 min (including all the resting period) and was conducted in a dimly lit soundproofed room.

#### 2.4.1. The original flankers task

In the original flankers task, each trial started with a fixation cross appearing at the center of the screen, and participants were asked to fix their attention on this cross. After 500 ms, the fixation cross disappeared, and the target and flanker characters were simultaneously presented for 150 ms, followed by a 2,000 ms blank screen. Participants were required to focus on the foveal target character and indicate whether they knew this character by pressing either “F” or “J” on the keyboard using their left and right index fingers. Half of the participants were instructed to press “F” if they knew the target character, while the other half were instructed to press “F” if they did not. Participants were instructed to respond as accurately and quickly as possible.

The experimental trials across all the five conditions (three conditions for lexical characters and two conditions for non-lexical characters, see Table 1) were pseudo-randomly displayed with the following constraints: (a) The same target character was not consecutively displayed. (b) Target characters of the same lexicality (lexical or non-lexical) were displayed consecutively at most three times. (c) The no-flanker trials (irrespective of lexical or non-lexical) were displayed consecutively at most three times. The total 720 trials were evenly divided into three blocks, with a short break between each block. Prior to the experimental trials, participants completed a practice block of 20 trials to familiarize themselves with the procedure.

#### 2.4.2. The revised flankers task

The revised flankers task differed from the original flankers task in the following way: each trial started with a central fixation cross for 500 ms. Then the target and flanker characters were simultaneously presented for 12 ms, followed by a blank ISI with duration set to the lexical decision threshold determined by the staircase procedure (see below). After the ISI, a post-mask composed of six “#” (######) was presented for 2,000 ms.

#### 2.4.3. The staircase procedure

Before running the revised flankers task, each participant underwent a staircase procedure to estimate the ISI threshold for the lexical decision. All the target characters in this procedure were presented without flanker characters. Each trial began with a central fixation cross for 500 ms. The target character was then flashed for 12 ms, followed by a blank ISI. After the ISI, a post-mask composed of six “#” (######) was presented for 2,000 ms, followed by a response interface (“请按键,” which means “please press the button”). Participants were instructed to respond to the target character as soon as they saw the response interface. The next trail started after participants responded.

Two staircases ran independently, starting at ISIs of 120 ms and 6 ms, respectively. The ISI changed following a 3-down, 1-up rule (decreased one step after three successive correct responses and increased one step after each incorrect response), and the step size up was always 4/3 of the step size down, making the staircase converge on the 83% correct threshold (Garcia-Perez, 1998). The step size (step size down) was initially set to 36 ms and decreased to 18 ms after two reversals, then to 6 ms after the next two reversals. Each staircase continued until it had reversed direction 16 times, and the ISI threshold was the mean value across the last ten reversals. The order of the two staircases was counterbalanced across participants. For each participant, the final estimated ISI threshold was calculated as the mean value of the results of the two staircases. Results of the staircase procedure for each participant were presented in Table 2 in Supplementary material. Two hundred target characters (100 lexical and 100 non-lexical characters) were used in the staircase procedure, which were not used in the flankers tasks. The target characters used in the staircase procedure and those used in the two flankers tasks were matched in terms of character frequency and visual complexity (indexed by stroke number).

### 2.5. Data analysis

Trials with incorrect responses were excluded from the reaction time (RT) analysis. Moreover, trials in each circumstance (lexical-original, lexical-revised, non-lexical-original, non-lexical-revised) with RT shorter than 100 ms or deviated more than 2.5 standard deviations (SDs) from the corresponding grand mean across all the participants were excluded from both the RT and accuracy rate (AR) analyses. Experimental effects were estimated using the lme4 package (Bates et al., 2015) in the R environment (Team, RC, 2022). Linear mixed-effect models (LMM), fitted with *lmer* function, were used to analyze RTs, and Logistic LMM models, fitted with *glmer* function, were used to analyze ARs. Only the successfully converged maximal random effects structures were included in all the analyses. The *p*-values were calculated using the lmerTest package (Kuznetsova et al., 2017). We reported regression coefficients (b), original errors (SEs), t-values (for RT analysis) or z-values (for AR analysis), and *p*-values. Fixed effects were deemed reliable if |t| or |z| values were beyond 1.96 (Baayen et al., 2008).

## 3. Results

### 3.1. Original flankers task

#### 3.1.1. Lexical target characters

For the analysis of RTs, the structure that converged included by-item random slopes and random intercepts, as well as only by-participant random intercepts. For the analysis of ARs, the structure that converged included only by-item and by-participant random intercepts.

**Figure 2:**
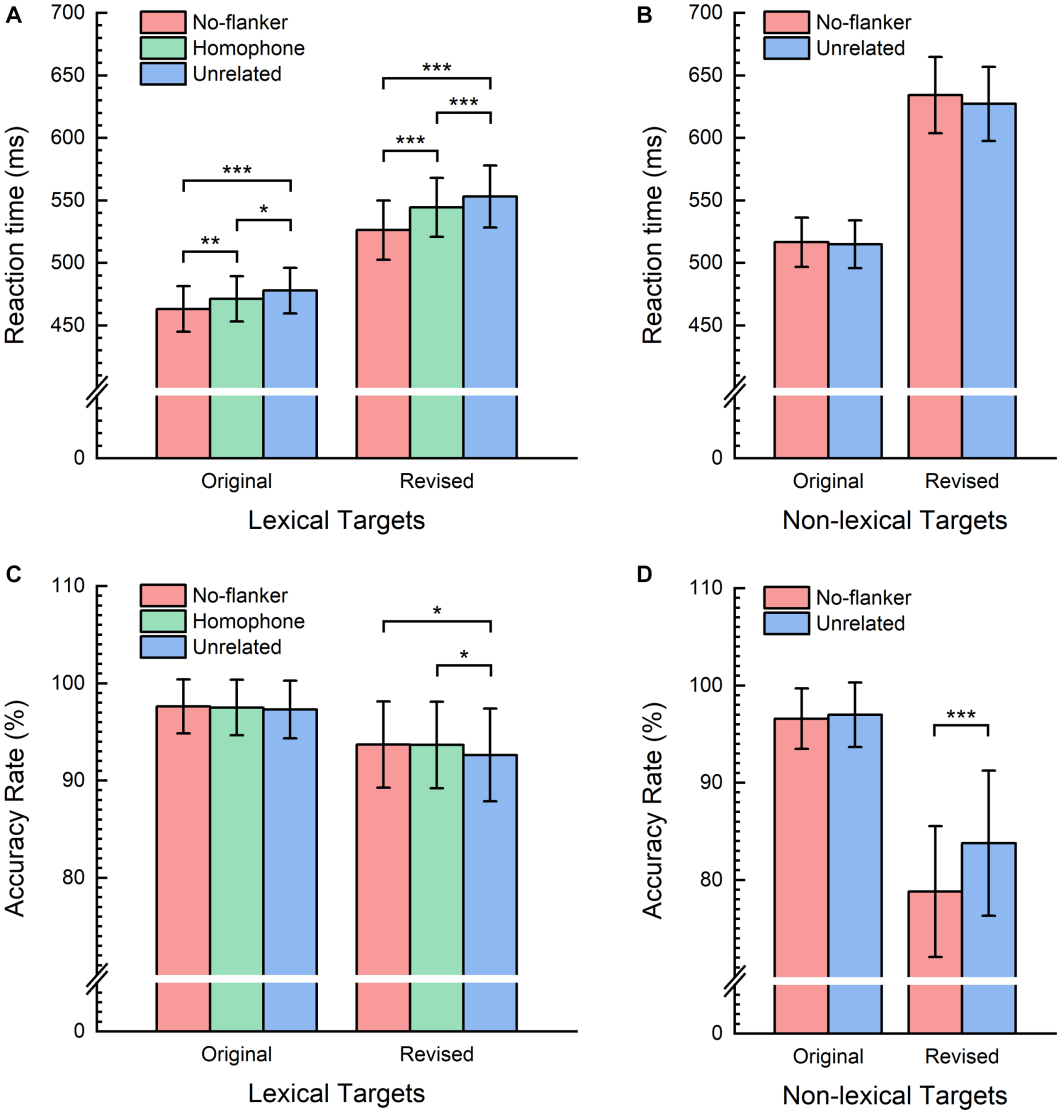
Histograms of the mean reaction times (RTs) and accuracy rates (ARs). **(A)** Mean RTs for the lexical target characters. **(B)** Mean RTs for the non-lexical target characters. **(C)** Mean ARs for the lexical target characters. **(D)** Mean ARs for the non-lexical target characters. Error bars represent one standard error mean. Asterisks denote two-tailed *p*-values obtained from bootstrapping: ***p < 0.001; **p < 0.01; *p < 0.05.

A total of 267 trials (2.5% of the total 10,800 trials) with incorrect responses were excluded from the RT analysis. Additionally, 274 outlier trials (2.5% of the total 10,800 trials) were removed from both the RT and AR analyses. Figures 2A,C present the mean RT and AR per condition, respectively. The mean RT and AR for each condition were as follows: 463.19 ms and 97.62% for the no-flanker condition, 471.37 ms and 97.51% for the homophone-flanker condition, and 477.89 ms and 97.30% for the unrelated-flanker condition. Planned pairwise comparisons were conducted across the no-flanker, homophone-flanker, and unrelated-flanker conditions. Results showed that: (1) the mean RT in the homophone-flanker condition was significantly shorter than that in the unrelated-flanker condition (*b* = −7.34, *SE* = 3.37, *t* = −2.18, *p* = 0.029), and the mean ARs were not significantly different between the two conditions (*b* = 0.09, *SE* = 0.14, *z* = 0.59, *p* = 0.555); (2) the mean RT in the homophone-flanker condition was significantly longer than that in the no-flanker condition (*b* = 8.20, *SE* = 2.97, *t* = 2.76, *p* = 0.006), and the mean ARs were not significantly different between the two conditions (*b* = −0.05, *SE* = 0.15, *z* = −0.33, *p* = 0.741); (3) the mean RT in the unrelated-flanker condition was significantly longer than that in the no-flanker condition (*b* = 15.53, *SE* = 2.70, *t* = 5.75, *p* < 0.001), and the mean ARs were not significantly different between the two conditions (*b* = −0.14, *SE* = 0.15, *z* = −0.92, *p* = 0.358).

#### 3.1.2. Non-lexical target characters

For the analysis of RTs, the structure that converged included by-item random slopes and random intercepts, as well as only by-participant random intercepts. For the analysis of ARs, the structure that converged included only by-item and by-participant random intercepts.

Three hundred thirty-four trials (3.1% of the total 10,800 trials) with incorrect responses were excluded from the RT analysis, and an additional 276 outlier trials (2.5% of the total 10,800 trials) were removed from both the RT and AR analyses. Figure 2B shows the mean RT for each condition, and Figure 2D shows the mean AR for each condition. The mean RT and AR for each condition were as follows: 516.61 ms and 96.59% for the no-flanker condition, and 514.90 ms and 96.99% for the unrelated-flanker condition. Our analyses did not reveal any statistically significant differences between the unrelated-flanker condition and the no-flanker condition for either the RTs (*b* = −1.80, *SE* = 2.44, *t* = −0.74, *p* = 0.459) or the ARs (*b* = 0.14, *SE* = 0.11, *z* = 1.22, *p* = 0.222).

### 3.2. Revised flankers task

#### 3.2.1. Lexical target characters

For the analysis of RTs, the structure that converged included by-participant random slopes and random intercepts, as well as only by-item random intercepts. For the analysis of ARs, the structure that converged included only by-item and by-participant random intercepts.

We excluded 702 trials (6.5% of the total 10,800 trials) with incorrect responses from the RT analysis, and an additional 306 outlier trials (2.8% of the total 10,800 trials) from both the RT and AR analyses. Mean RT and AR per condition are presented in Figures 2A,C, respectively. The mean RT and AR for each condition were as follows: 525.26 ms and 93.69% for the no-flanker condition, 544.54 ms and 93.66% for the homophone-flanker condition, and 553.20 ms and 92.63% for the unrelated-flanker condition. Planned pairwise comparisons were conducted across the no-flanker, homophone-flanker, and unrelated-flanker conditions. Results showed that: (1) the mean RT in the homophone-flanker condition was significantly shorter than that in the unrelated-flanker condition (*b* = −9.92, *SE* = 2.87, *t* = −3.46, *p* < 0.001), and the mean ARs was significantly higher in the homophone-flanker condition compared with the unrelated-flanker condition (*b* = 0.19, *SE* = 0.10, *z* = 2.00, *p* = 0.045); (2) the mean RT in the homophone-flanker condition was significantly longer than that in the no-flanker condition (*b* = 17.40, *SE* = 3.73, *t* = 4.66, *p* < 0.001), and the mean ARs were not significantly different between the two conditions (*b* = −0.0002, *SE* = 0.10, *z* = −0.002, *p* = 0.998); (3) the mean RT in the unrelated-flanker condition was significantly longer than that in the no-flanker condition (*b* = 27.31, *SE* = 4.34, *t* = 6.29, *p* < 0.001), and the mean AR was significantly lower in the unrelated-flanker condition compared with that in the no-flanker condition (*b* = −0.19, *SE* = 0.10, *z* = −2.00, *p* = 0.045).

#### 3.2.2. Non-lexical target characters

For the analysis of RTs, the structure that converged included by-participant random slopes and random intercepts, as well as only by-item random intercepts. For the analysis of ARs, the structure that converged included only by-item and by-participant random intercepts.

We excluded 1898 trials (17.6% of the total 10,800 trials) with incorrect responses from the RT analysis, and additional 285 outlier trials (2.6% of the total 10,800 trials) were removed from both the RT and AR analyses. The results are displayed in Figures 2B,D, which show the mean RT and AR for each condition. The mean RT and AR for each condition were as follows: 634.26 ms and 83.78% for the no-flanker condition, and 627.30 ms and 78.79% for the unrelated-flanker condition. Our analyses revealed no significant difference in RTs between the unrelated-flanker and the no-flanker conditions (*b* = −5.44, *SE* = 5.33, *t* = −1.02, *p* = 0.307). However, we did observe a significant difference in ARs: the unrelated-flanker condition showed a higher accuracy rate than the no-flanker condition (*b* = 0.40, *SE* = 0.06, *z* = 6.98, *p* < 0.001).

## 4. Discussion

The current study used the original and the revised flankers tasks to investigate the parallel phonological processing of Chinese characters. In both tasks, we consistently observed the significant phonological POF effect reflected by longer reaction times to the lexical targets in the unrelated-flanker condition compared to the homophone-flanker condition (Figure 2A), suggesting parallel phonological processing of Chinese characters. In contrast, previous research did not observe the phonological POF effect in the flankers task using alphabetic scripts (Cauchi et al., 2020). The discrepancy is likely due to the difference in orthography between alphabetic and logographic scripts (orthography-phonology association vs. orthography-meaning association).

### 4.1. Parallel phonological processing of Chinese characters

In both the original and the revised flankers tasks, we observed the significant phonological POF effect. In the revised flankers task, even though the exposure of the stimulus was strictly controlled that participants were unable to process the target and flanker characters serially, the phonological information of the flankers would influence participants’ reaction times and accuracy rates to the targets, providing reliable evidence for the parallel phonological processing of Chinese characters.

Previous studies have explained that the POF effects at orthographic, semantic and syntactic levels in flankers tasks were due to the spatial integration of information across the target and flankers (Dare and Shillcock, 2013; Snell et al., 2017a,b,c, 2021; Snell and Grainger, 2019). For instance, if the shared orthographic information from the target and the flankers (e.g., ro rock ck) was activated in parallel, this shared information would be spatially integrated, leading to a spatial priming effect that facilitated participants’ lexical decisions to the target words (Dare and Shillcock, 2013). Results of the present study extended this perspective by that the phonological information from parafoveal and foveal characters could also be spatially integrated. However, we contend that how the spatial integration of information facilitates lexical decisions to the targets needs more detailed explanation. Here we propose a “phonological threshold hypothesis” to explain the phonological POF effect observed in our study. This hypothesis is derived from the Lexical Constituency Model (Perfetti et al., 2005), which proposes that there are three levels during visual word recognition: orthography, phonology, and semantics. At each level, there are multiple units. For instance, at the phonological level in Chinese, each syllable is coded by the combination of three types of units (onset, vowel, and tone). The Lexical Constituency Model proposes that there is an activation threshold for each unit at all three levels. Before reaching that threshold, the units at one level (e.g., phonology) cannot send output to the units at another level (e.g., semantics). Therefore, to access the semantic meaning of a character, there should be sufficient activation of the corresponding phonological units to reach the phonological threshold. According to the Lexical Constituency Model, in the current study, the target character and its homophone flankers activated the same phonological units in parallel, so for the target character, there were more phonological inputs in the homophone-flanker condition compared with those in the unrelated-flanker condition. Thus, in the homophone-flanker condition, it is easier for the target to reach its phonological threshold and activate its phonological information. We conjecture that since non-lexical targets were all unpronounceable, participants would make lexical decisions once the phonological information of the lexical targets was activated. Therefore, compared to the unrelated-flanker condition, participants could make significantly faster lexical decisions in the homophone-flanker condition where the phonological information of the targets was easier to activate. Notably, this threshold hypothesis could also explain the orthographic and semantic POF effects observed in the previous studies using flankers tasks (Dare and Shillcock, 2013; Grainger et al., 2014; Snell et al., 2017a, 2018a, 2019, 2021; Meade et al., 2021). Specifically, the flankers that shared orthographic or semantic information with the target would make the target reach its orthographic threshold or semantic threshold easier, leading to faster target recognition.

The phonological POF effect observed in our study has implications for the models of reading. For instance, recently, an integrated model of word processing and eye-movement control during Chinese reading, known as the Chinese Reading Model (CRM), was proposed (Li and Pollatsek, 2020). This model assumes that all characters within the perceptual span are processed in parallel. However, because the CRM does not include either a phonological processing component or a semantic processing component, it remains unclear whether the parallel processing of Chinese characters, as proposed by CRM, refers to parallel orthographic, phonological, or semantic processing. The results of the present study suggest that multiple characters can be processed in parallel at least to the phonological level, which are valuable for including a phonological processing component in the CRM.

Previous eye-tracking studies have used Chinese characters as stimuli to investigate the phonological POF effect in sentence reading. One study reported that beginning Chinese readers showed a significant phonological POF effect (Zhou et al., 2018), and another study observed a phonological POF effect in proficient readers using an oral reading task (Pan et al., 2016). These findings suggest that there is parallel phonological processing of Chinese characters in sentence reading. Nevertheless, there were two studies which did not observe the phonological POF effect in silent Chinese reading (Yan et al., 2009; Pan et al., 2016). The inconsistent results may stem from the possibility that the eye-tracking technique lacks the sensitivity to reveal the parallel phonological processing of Chinese characters.

### 4.2. Attentional mechanism in parallel processing of visual words or Chinese characters

The present study observed a significant effect of flanker presence on target recognition. In both flankers tasks, for the lexical target characters, reaction times were significantly shorter when the target characters were presented in isolation (no-flanker condition) compared to the other two with-flanker conditions (homophone-flanker and unrelated-flanker conditions) (Figure 2A). This result is consistent with a previous study in alphabetic scripts, which also reported the effect of flanker presence in the flankers task (Snell et al., 2017a). The effect of flanker presence suggests that a portion of attentional resources shifted to the flankers in the with-flanker conditions, and because participants focused their attention on the target characters, the attention distribution to the flankers was likely a covert and inevitable process. Moreover, another study found that in the flankers task, participants’ pupil size was contingent with the brightness of the locations of the horizontally aligned flanker words (Snell et al., 2018b). This finding further suggests the attention distribution over both the target and flanker words, as the pupil light response could index participants’ covert attention to certain locations (Mathot et al., 2013).

How attention is allocated during reading is a fiercely debated question and there are competing views between the sequential attention shift (SAS) models and the parallel processing gradient (PG) models. The SAS models such as the E-Z reader model propose that during reading, attention is confined to only one word at a time (Reichle et al., 2003, 2006, 2009, 2011), whereas the PG models such as the SWIFT and the OB1-reader models maintain that during reading, attention is distributed over multiple words (Engbert et al., 2005; Richter et al., 2006; Snell et al., 2018c). The effect of flanker presence found in our study and the previous study (Snell et al., 2017a) supports the attention distribution over multiple words proposed by the PG models.

For the non-lexical target characters, in both flankers tasks, we did not observe the effect of the flanker presence (Figure 2B): participants’ reaction times showed no significant difference between the unrelated-flanker and the no-flanker conditions. This was likely because the unrelated flanker characters facilitated lexical decisions to the non-lexical targets compared to the no-flanker condition, canceling out the effect of attention distribution to the flankers. This facilitation effect was evident from the significantly higher accuracy rate in the unrelated-flanker condition compared to the no-flanker condition in the revised flankers task (Figure 2D). However, whether the absence of an RT difference in the non-lexical conditions truly supports attention distribution to the flankers remains a question that requires further research.

### 4.3. The role of phonology in Chinese character recognition

The phonological POF effect observed in the present study indicates that phonological information is activated automatically during Chinese character recognition. The role of phonology in Chinese character recognition is a long-standing debate. Some studies have suggested that phonology does not play an essential role during Chinese character recognition and Chinese characters are recognized through the direct access to semantics via orthography (Zhou and Marslen-Wilson, 1996; Chen and Shu, 2001; Wang et al., 2010; Wong et al., 2014; Zhang et al., 2019). For instance, a study using the masked-priming paradigm found null priming effects in the pure phonological-related prime condition compared to the character-related and semantic-related prime conditions, suggesting that phonological activation was not mandatory in Chinese word recognition (Wong et al., 2014). In contrast, some other studies have suggested that phonological information is automatically activated and plays an important role during Chinese character recognition (Perfetti and Zhang, 1995; Chua, 1999; Xu et al., 1999; Spinks et al., 2000; Guo et al., 2005; Li et al., 2013). For instance, phonological preview benefits have been reported using Chinese characters as stimuli, indicating the involvement of phonological codes in character recognition (Pollatsek et al., 2000; Liu et al., 2002; Tsai et al., 2004). In the present study, we used the lexical decision task in which participants were asked to fixate their eyes on the target character and ignore the flankers. Even under such circumstances, the phonological information of the lexical parafoveal flankers was activated and influenced the processing of the lexical target characters. This finding suggests that the phonological information is not only automatically activated but also contributes to Chinese character recognition.

### 4.4. Parallel phonological processing of alphabetic scripts

As introduced, Cauchi’s study did not observe the phonological POF effect in the flankers task in alphabetic scripts (Cauchi et al., 2020). In contrast, our study observed this effect using logographic scripts (Chinese). Therefore, the absent phonological POF effect in the flankers task in alphabetic scripts was unlikely due to the postulation that phonological processing of a parafoveal word was tied to its specific spatial location and not integrated into the central channel for word recognition (Grainger et al., 2014; Snell et al., 2017b; Cauchi et al., 2020). Notably, the phonological POF effect observed in the present study was quite small (a 7 ms effect in the original flankers task and a 9 ms effect in the revised flankers task when contrasting the results between the homophone-flanker and unrelated-flanker conditions). Therefore, for alphabetic scripts, there might also be a small phonological POF effect, but the phonological overlap between the target words and the orthographic-control pseudowords diluted this effect to the point that it could not be observed (see Introduction). However, since logographic scripts differ markedly from alphabetic scripts, whether the findings in our study reveal the universality of parallel processing of written scripts or the unique mechanisms underlying the processing of logographic scripts requires further exploration.

### 4.5. The original flankers task versus the revised flankers task

As introduced, the revised flankers task addressed two concerns regarding the original flankers task: long stimulus duration and a lack of post-masks. Therefore, we contend that the revised flankers task is more rigorous than the original one for investigating the parallel processing of visual words. In the present study, both the original and the revised flankers tasks reveal the parallel phonological processing of Chinese characters. However, due to the two experimental concerns of the original task, the results of the revised task are more reliable evidence. Moreover, in the original task, because the sufficient stimulus exposure time (150 ms) allowed participants to recognize each target character in each condition accurately, accuracy rates in the three lexical conditions (no-flanker, homophone-flanker, unrelated-flanker) were of no significant difference and were all close to 100% (Figure 2C). However, in the revised task for lexical targets, the accuracy rate in the unrelated-flanker condition was significantly lower compared to that in the other two conditions (homophone-flanker and no-flanker conditions) (Figure 2C). This was likely because in the revised task, the blank ISI was set to each participant’s lexical decision threshold, based on the results of the “no-flanker trials” in the staircase procedure. As a result, there would be more trials in the unrelated-flanker condition where participants could not recognize the targets due to inadequate stimulus exposure time (since participants exhibited the longest recognition times for the targets in this condition). This would cause a lower accuracy rate in the unrelated-flanker condition compared to the other two conditions in which participants could recognize the target characters more rapidly. The significantly higher accuracy rate in the homophone-flanker condition compared to the unrelated-flanker condition further supports the parallel phonological processing of Chinese characters, and suggests that the phonological POF effect does not solely arise from a speed-accuracy trade-off.

For the non-lexical targets, the accuracy rate showed no difference between the no-flanker and unrelated-flanker conditions in the original task due to the sufficient stimulus exposure time (150 ms). In contrast, in the revised task, the accuracy rate was significantly higher in the unrelated-flanker condition than in the no-flanker condition, an outcome likely attributed to the experimental design. In the revised task, characterized by an exceedingly brief stimulus exposure time, participants encountered challenges in recognizing the lexicality of each target accurately. In the unrelated-flanker condition, participants could notice that the target and the flankers were always of the same lexicality. As a result, the parallel processing of the target and the flankers might help participants make correct lexical decisions. Specifically, participants could make correct lexical decisions regarding the non-lexical targets even if they only recognized the flankers but not the target as non-lexical. Therefore, for the non-lexical targets, the accuracy rate was significantly higher in the unrelated-flanker condition than in the no-flanker condition.

### 4.6. Flankers tasks and natural reading

Lastly, one may argue that the flankers task is distinct from natural reading. Therefore, based on the results of the current study, no inference can be made about whether there is parallel phonological processing in natural reading. Nonetheless, some studies have suggested that similar mechanisms are involved in the flankers task and natural reading. For instance, applying the flankers task, while attention was found to be biased to the left using non-linguistic stimuli (Harms and Bundesen, 1983; Hommel, 1995), the attention was found to have a rightward bias using words as stimuli (Snell and Grainger, 2018), which is analogous to natural reading. Moreover, the POF repetition (the words in the fovea and parafovea are the same) effects in both the flankers and natural reading tasks were reflected by a negatively deflected ERP component that began around 200–250 ms after stimulus/fixation onset and that persisted into the N400 time-window, and the effects both exhibited a broad distribution across the scalp (Snell et al., 2019; Mirault et al., 2020). These ERP results imply that the effects observed in the flankers task result from the mechanisms similar to those driving the effects in natural reading tasks. Therefore, the flankers task is a valuable tool for exploring the parallel processing of linguistic information, and the findings obtained from the flankers task would provide valuable insights into the mechanisms involved in natural reading.

## 5. Conclusion

In sum, the current study indicates that readers can process the phonological information from foveal and parafoveal Chinese characters simultaneously, extending the empirical findings that reveal the parallel processing mechanisms underlying reading. Moreover, results of the current study suggest that phonological information is automatically accessed and contributes to Chinese character recognition.

## Data availability statement

The original contributions presented in the study are included in the article/Supplementary material, further inquiries can be directed to the corresponding author.

## Ethics statement

The studies involving humans were approved by the Ethics Committee of Sichuan University. The studies were conducted in accordance with the local legislation and institutional requirements. The participants provided their written informed consent to participate in this study.

## Author contributions

RY designed the study. RY and YW collected and analyzed the data, and wrote the original manuscript. FG reviewed and edited the manuscript. All authors contributed to the article and approved the submitted version.

## Footnotes

^1^ https://deepai.org/machine-learning-model/image-similarity
